# Providing outpatient cancer care for CALD patients: a qualitative study

**DOI:** 10.1186/s13104-021-05724-3

**Published:** 2021-08-09

**Authors:** Bróna Nic Giolla Easpaig, Yvonne Tran, Teresa Winata, Klay Lamprell, Diana Fajardo Pulido, Gaston Arnolda, Geoff P. Delaney, Winston Liauw, Kylie Smith, Sandra Avery, Kim Rigg, Johanna Westbrook, Ian Olver, David Currow, Afaf Girgis, Jonathan Karnon, Robyn L. Ward, Jeffrey Braithwaite

**Affiliations:** 1 Australian Institute of Health Innovation, Macquarie University, Level 6, 75 Talavera Road, North Ryde, NSW 2109 Australia; 2 Infant, Child and Adolescent Mental Health Services, Liverpool Hospital, Liverpool, NSW Australia; 3 South-Western Sydney Local Health District, Liverpool, NSW Australia; 4 South Western Sydney Clinical School, University of New South Wales, Liverpool, NSW Australia; 5 Ingham Institute for Applied Medical Research, Liverpool, NSW Australia; 6 St. George Cancer Care Centre, St. George Hospital, Kogarah, NSW Australia; 7 St. George Hospital Clinical School, University of New South Wales, Sydney, NSW Australia; 8 Faculty of Health and Medical Sciences, University of Adelaide, Adelaide, SA Australia; 9 College of Medicine and Public Health, Flinders University, Adelaide, SA Australia; 10 Faculty of Health, University of Technology Sydney, Sydney, NSW Australia; 11 Prince of Wales Clinical School, University of New South Wales, Sydney, NSW Australia; 12 Faculty of Medicine and Health, The University of Sydney, Sydney, NSW Australia

**Keywords:** Multidisciplinary care, Cancer outpatient, Culturally and linguistically diverse, CALD, Minority, Ambulatory care, Patient-centred, Ethnography, Qualitative

## Abstract

**Objective:**

There have been few descriptions of how outpatient cancer care is provided to patients from culturally and linguistically diverse (CALD) communities. As populations who experience disparities in cancer care access and outcomes, deeper understanding is needed to help identify those factors which can shape the receipt of multidisciplinary care in ambulatory settings. This paper reports on data collected and analysed as part of a multicentre characterisation of care in Australian public hospital cancer outpatient clinics (OPCs).

**Results:**

Analysis of data from our ethnographic study of four OPCs identified three themes: “Identifying CALD patient language-related needs”; “Capacity and resources to meet CALD patient needs”, and “Making it work for CALD communities.” The care team comprises not only clinicians but also families and non-clinical staff; OPCs serve as “touchpoints” facilitating access to a range of therapeutic services. The findings highlight the potential challenges oncology professionals negotiate in providing care to CALD communities and the ways in which clinicians adapt their practices, formulate strategies and use available resources to support care delivery.

**Supplementary Information:**

The online version contains supplementary material available at 10.1186/s13104-021-05724-3.

## Introduction

How is multidisciplinary cancer care provided to patients from culturally and linguistically diverse (CALD) communities within ambulatory cancer settings? Multidisciplinary care is considered best practice in cancer care [1–4]. It seeks to promote equitable, evidence-based care by a multidisciplinary team (MDT) that combines expertise relevant to the disease and to patient needs, and supports patients in decision-making about their care [5, 6]. Research indicates that people with cancer may have a variety of needs including medical, informational and supportive care needs [7–9]. Hospital outpatient clinics (OPCs), are central sites which can support collaboration between different areas of expertise (such as allied health professionals) to better meet these patient needs.

Multidisciplinary care can be challenging to accomplish, given the complexities of cancer and its management, with multiple transitions between service providers and the burden of illness experienced by patients [10–13]. For those in CALD communities, a variety of factors can detrimentally impact healthcare access and experience, including: language and communication issues [10, 14–20]; cultural attitudes and beliefs about illness [17, 21–23]; poorer health literacy[21] anticipated or experienced cultural insensitivity, discrimination, lack of cultural competence on the part of providers [17]; and sub-optimal access to, or use of interpreter services [14, 16–19, 21, 23]. In oncology, studies indicate that providers often adopt a variety of strategies to aid communication with CALD patients and their families [15]. However, concerns have been raised about the quality and sufficiency of information provision and support where language barriers exist [15, 18, 23, 24].

A deeper understanding of care provision for CALD communities is needed to identify those factors which may contribute to disparities in cancer outcomes, to inform service improvement [7]. We conducted a multi-centre study which characterised the organisation and practice of care in outpatient clinics (OPCs) and identified the barriers to, and facilitators of its provision. This paper reports the CALD-relevant data collected as part of that study [25].

## Main text

### Methods

A multi-site ethnography was conducted in four public hospital cancer OPCs over a 9-month period [26, 27]. This ethnographic method was selected to provide an in-depth and sophisticated examination of health care settings [26, 27]. Specifically, this approach comprised of: in-situ observations so to generate descriptions of the nuanced day to day reality of care delivery in OPCs; interviews with key professionals in order to provide insights into care processes and learn about barriers and facilitators to this work; and document review to allow consideration of the aforementioned data within the relevant service provision context.

#### Context of services

Hospitals were located across two government healthcare districts that serve as hubs, drawing together networks of multidisciplinary professionals including doctors, nurses and allied health professionals from all disciplines related to oncology care to facilitate professional collaboration, patient referral and impromptu consultations. An overview of services in these areas is shown in Table 1.

**Table 1:** Overview of services at hospital study sites

Health district details	Setting	OPC occasions of service	Key services available
District 1- is an area with a population of 966,450, with 4738 new cases diagnosed in 2017, and consists of outer metropolitan and regional locations	Service 1	9630 Occasions of service provided in 2016/17	Chemotherapy Care coordination Palliative care coordination Allied health Oncology pharmacy Assessment spaces On-site multidisciplinary team meetings (limited range)
	Service 2	40,624 Occasions of service provided in 2016/17	Chemotherapy Radiation treatment Care coordination Palliative care coordination Allied health Oncology pharmacy Assessment spaces On-site multidisciplinary team meetings (limited range)
	Service 3	93,853 Occasions of service provided in 2016/17	Chemotherapy Radiation treatment Care coordination Palliative care coordination Allied health Oncology pharmacy Assessment spaces On-site multidisciplinary team meetings
District 2-is an area with a population of over 900,000, with 4,912 new cases diagnosed in 2017, and consists of outer metropolitan and regional locations	Service 4	10,400 occasions of service provided between Jul 2010–Jun 2011	Chemotherapy Care coordination Palliative care coordination Allied health Oncology pharmacy Assessment spaces

Source: Information obtained via plans, reports, and observations

In both districts, well over one third of residents were born overseas, almost half of residents in district 1 and over one third of district 2 residents spoke a language other than English at home and approximately 10% of residents in district 1 and 6% in district 2 reported speaking English ‘not well or not at all’; all higher than the reported averages for NSW [28]. Other than English, languages commonly spoken in these districts were Arabic, Vietnamese, Mandarin, Cantonese and Greek.

#### Procedure

*Recruitment:* Professional groups targeted for interviews were recruited using email and on-site information sessions; informed consent was obtained. Permission to observe was granted by authorities responsible for OPCs.

*Fieldwork:* A range of data were collected during in-situ observation (n = 135 h approximately), key informant interviews (n = 13) (please see Additional file 1 for interview guide) and document review (n = 8), as detailed in Table 2.

**Table 2:** Fieldwork details

Method	Key areas of focus	Sources of data	Documentation details
Non-participant observations and informal discussions	The delivery of multidisciplinary care including observation of planned and ad hoc collaborations of relevant staff	Observations were undertaken in chemotherapy treatment areas, waiting rooms, consultation spaces and other multipurpose areas of OPCs (not within consultation rooms). Nursing, care coordination, allied health, medical, clinical research, administrative and management professional groups were observed (n = 135 h approximately)	Observations and reflections were recorded in 312 pages of handwritten fieldnotes and then transcribed
Key informant interviews	The organisation of practice and models of care used, complexity of needs, as well as barriers and facilitators to practice	Care coordinators, tumour-specific specialist nurses, cancer nurse specialists and senior clinical staff were interviewed (n = 13), 6.5 h	Interviews took on average 30 min and were audio-recorded and transcribed (54,124 words)
Document collection	The current context of service provision, service planning, models of care and guidelines for professional practice	Documents were sourced online and via consultation with the services (reviewed for 15 h)	Documents were collated and reviewed on an ongoing basis (n = 8)

*Data management:* Data were managed securely and analysed using an inductive approach, as outlined elsewhere [29–31]. From this corpus of data, a sub-set relevant to care for CALD patients was extracted, collated, and a coding framework developed. Face validity of the framework was achieved via review from experienced team members (BNGE, KL and DFP) and data were thematically analysed [31].

### Results

**Fig. 1:**
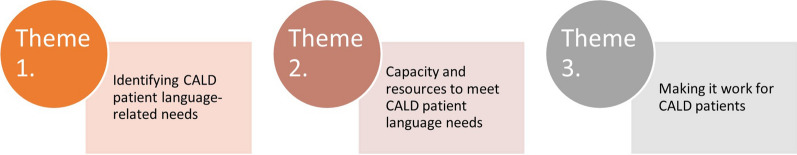
Analytic themes for the provision of outpatient care to CALD patients

Analysis resulted in three themes shown in Fig. 1.

#### Theme 1. Identifying CALD patient language-related needs

Oncology staff were keenly aware they were providing care for CALD populations and reflected upon this positively:

“I quite like the challenge that comes with working in this district. Different cultures, everyone has a different story to tell.” (Key Informant (KI6))

Staff also reported this could be a “struggle” (KI6). They appreciated that CALD patient needs may be more complex to identify and address, with implications for the coordination of care and engagement with other professionals.

A chief focus in OPCs was the identification of patients for whom language was a barrier. Staff considered patients’ English language proficiency and the support available from family and friends when determining needs. It was not always possible to identify these patients in advance:

“…frequently someone will just come up and say [something in another language], and the most I can say is ‘what language?’ They might say ‘Vietnamese’ and then I'll have to call the interpreter service to get someone on the phone.” (KI8)

Where oncology staff were aware of language barriers, this alerted them to the need for written information in the patient’s primary language, allowing additional time for consultations and the support of interpreter services:

“We need to organise that for them and to have them present to sign the consent form for treatment. If there isn’t a physical interpreter present in the consultation, then most likely we might use a telephone interpreter.” (KI4)

Identifying and addressing these needs was crucial for establishing shared understandings and facilitating consent-giving processes.

#### Theme 2. Capacity and resources to meet CALD patient language needs

Generally, in hospitals, professional health care-specific interpreters (HCIs) and multilingual signage were available. Two cancer OPCs offered relevant written multilingual resources and in one OPC, a multilanguage electronic check-in system was available. However, not all resources were easily-accessible for CALD patients, and challenges were reported by staff:

“I do a lot of my consults on the phone. It’s [the symptom screening tool tool] really lengthy… to sit there on the phone and [talk with] patients that don’t speak English very well. I don’t think it is working very well.” (KI7)

Some staff were able to communicate with patients and their families in a range of languages and multilingualism was highly valued. One staff member reflects:

“One of the big challenges for me is not being able to speak Vietnamese or Arabic.” (KI5)

Staff expressed appreciation for the work of HCIs and viewed this service as vital. Nonetheless, difficulties were noted concerning service availability, especially when support was required spontaneously:

“It takes about 20 minutes for them to even answer the phone [referring to the HCI service], and then to get someone who is available … It just happened to me yesterday. [It] ended up being like a three-way call between the patient and the daughter and the interpreters and back and forth between them; … multiple phone calls … for a simple query from the patient; it was a two hour affair … because it was so hard to get them.” (KI1)

Staff noted a preference for in-person interpretation because of the difficulties associated with the telephone-mediated service. They described the telephone service as hindering communication with the patient and more “time-consuming.” (KI3).

#### Theme 3. Making it work for CALD patients

Oncology staff employed various strategies to help bridge the gap between identified needs and the resources available. As staff depend on the availability of HCIs, they reported rescheduling or staying later to access them. They also prioritised and reorganised workload as proactive strategies to maximise the likelihood of accessing in-person interpretation:

“If there is a known psychosocial issue, […] if we see them in clinic then we've got the interpreter. Where it is an English-speaking person, I know I can call them the next day and speak on the phone. So, I would prioritise the non-English speaking person [for the clinic].” (KI2)

Staff tailored their approaches based on their assessment of language proficiency. For example, some aspects of care could be discussed over the phone, while other aspects required a HCI-supported discussion at the clinic.

Securing interpreter services was a group-based activity in some OPCs:

Staff providing administrative support appeared to be regularly on the phone attempting to secure HCIs. Clinical and non-clinical staff alike were involved in obtaining these services for patients. (Fieldnote p26)

The care team expands to encompass professionals with the expertise necessary to meet patient needs and, for CALD patients, our data would suggest that not only HCIs and non-clinical staff but also family and friends could be considered part of this team. All these groups made contributions in the form of advocacy, interpretation/translation, as sources of information about cultural needs and as providers of emotional support.

### Discussion

The ethnographic exploration of multidisciplinary care in ambulatory settings enabled the generation of a detailed account of care delivery to CALD patients, informed by direct observations and insights of those on the frontlines of providing this care. Analysis of these data led to the identification of pertinent themes and behavioural examples of prioritization, strategizing, adaption and work reorganization in an attempt to provide the best care possible for CALD patients in day-to-day practice. Within the multidisciplinary care and patient-centred paradigm, patients are considered as partners in decision-making [32]; language and cultural barriers impede patient understanding and consent-giving [33]. Consistent with the literature, ensuring availability of HCIs, allowing additional time and providing translated resources [15–18, 21] were needed to support patient-provider interactions. Based on our observations and discussions, this appears to require additional resourcing to ensure that CALD patients receive the same care quality as the majority population. As such, we support calls to assess the additional cost of care to CALD populations so that they can be factored into budgetary provisions.

Studies have identified strategies used by oncology professionals to facilitate interactions with CALD patients, such as rapport-building and employing non-verbal modes of communication [15, 16]. In OPCs, staff anticipate issues and formulate strategies to mitigate the risk of ineffective interaction. Nominated strategies included prioritisation and reorganisation of workload and schedules to facilitate optimal engagement with CALD patients, and collaboration among clinical and non-clinical staff to obtain interpreter services.

The desire expressed for increased in-person interpreter services in the OPC may, if addressed, yield additional benefits. These settings function as hubs for a multidisciplinary range of professionals, including many categories of allied health staff. Concerns have been expressed that CALD patients may not receive sufficient or equitable psychosocial support, hope-giving or information [15, 18, 23, 24]. Shifting practice to ensure in-person, translator-supported OPC attendance could facilitate prompt identification of needs and access to relevant therapeutic services. In light of the large-scale uptake of telemedicine during the COVID-19 pandemic [34, 35], it is important to consider the implications for CALD patients, and to explore the possible role of videoconferencing with an interpreter, or integrated care with a General Practitioner who knows the patient and speaks their language.

Our findings suggest a need to conceptualise the multidisciplinary team in OPCs as a collaboration of clinical and non-clinical staff including HCIs, as well as the families of CALD patients. Emotional, informational and translation support and advocacy may be welcomed given the burden of illness and complexities of cancer care [10–13]. Clarity is needed about the specific contributions made by team members, as there can be a misunderstanding about the role of HCIs [19] (e.g., to translate vs. providing emotional support). Further, while family and friends can make important contributions to supporting patient care, it is not considered appropriate for them to act as HCIs, to ensure patient autonomy [16, 33, 36].

Consistent with the literature, capacity to accurately and comprehensively identify patients with linguistic needs within a timeframe sufficient to make appropriate arrangements and ensure the provision of translated resources was identified as helpful [15, 16, 19]. The findings support the view that oncology staff need interpreter services to be available and flexible, and prefer in-person translation services [15, 16]. Also observed were aspects of services which may enhance accessibility (e.g., multilanguage check-in kiosks). These findings highlight not only the potential challenges staff negotiate in OPCs, but the ways in which they adapt their practice, formulate strategies and use available resources to provide care to CALD patients.

There is a need to develop evidence-informed guidelines on how hospitals should optimise care delivery for CALD populations and the associated costs. Adherence with guidelines could be monitored, which could be linked to funding mechanisms, i.e., hospitals adhering to guidelines receive additional funding. Better data collection to routinely identify CALD patients and the expenditures related to their care, compared to a similar level of care provided to non-CALD patients, could be a basis for determining whether activity-based funding formula should include a loading for these patients [37] as occurs for Aboriginal and Torres Strait Islander patients [38]. Observing the additional complications for care-giving in the presence of language-barriers, it is crucial to estimate and provide for the additional resources required to provide equitable care.

## Limitations

This study opportunistically captured data about care to CALD patients within the broader study; there is a clear need for ethnographic research explicitly tailored to this population. The perspectives of CALD patients, their families and communities, and of HCIs, are needed to comprehensively study these issues. The study examined care provided to those patients who have been referred and are being treated at cancer centres, while evidence indicates reduced screening rates and delays in presentations for CALD community members [39, 40]; an important aspect of inequalities in cancer care not addressed in this paper. Overcoming language barriers was what was observed to dominate practice and is the focus of this paper; it is likely that some of the communication difficulties also reflect cultural differences [19, 23], which were not systematically explored.

## Supplementary Information

**Additional file 1.** Interview topic guide. A topic guide for the interviews with key informants.

## Data Availability

The datasets generated and analysed during the current study are not publicly available due to the conditions of ethical approval, however de-identified data are available from the corresponding author on reasonable request.

## Abbreviations

CALD: Culturally and linguistically diverse
OPC: Outpatient clinic

## References

[1] American College of Surgeons: Commission on Cancer (2016). Cancer program standards: ensuring patient-centered care.

[2] Jiang H (2015). Patients should surely be included in multidisciplinary team meetings in China healthcare. Response to Thornton, S (2015) Time to review utility of multidisciplinary team meetings. BMJ.

[3] Pan CC, Kung PT, Wang YH, Chang YC, Wang ST, Tsai WC (2015). Effects of multidisciplinary team care on the survival of patients with different stages of non-small cell lung cancer: a national cohort study. PLoS ONE..

[4] Saini KS, Taylor C, Ramirez AJ, Palmieri C, Gunnarsson U, Schmoll HJ (2012). Role of the multidisciplinary team in breast cancer management: results from a large international survey involving 39 countries. Ann Oncol.

[5] Cancer Australia. All about multidisciplinary care [Internet]. Strawberry Hills, NSW [updated 2017; ]. https://canceraustralia.gov.au/clinical-best-practice/multidisciplinary-care/all-about-multidisciplinary-care. Accessed 1 Oct 2020.

[6] Zorbas H, Barraclough B, Rainbird K, Luxford K, Redman S (2003). Multidisciplinary care for women with early breast cancer in the Australian context: what does it mean?. Med J Aust.

[7] Dilworth S, Higgins I, Parker V (2014). Patient and health professional's perceived barriers to the delivery of psychosocial care to adults with cancer: a systematic review. Psychooncology.

[8] Rankin NM, Barron JA, Lane LG (2011). Psychosocial oncology services in New South Wales. Aust Health Rev.

[9] Tran Y, Lamprell K, Nic Giolla Easpaig B (2019). What information do patients want across their cancer journeys? A network analysis of cancer patients' information needs. Cancer Med.

[10] Harun A, Harrison JD, Young JM (2013). Interventions to improve patient participation in the treatment process for culturally and linguistically diverse people with cancer: a systematic review. Asia Pac J Clin Oncol.

[11] Shaw T, York S, White K, McGregor D, Rankin N, Hawkey A (2018). Defining success factors to describe coordinated care in cancer. Transl Behav Med.

[12] Walsh J, Harrison JD, Young JM, Butow PN, Solomon MJ, Masya L (2010). What are the current barriers to effective cancer care coordination? A qualitative study. BMC Health Serv Res.

[13] Walsh J, Young JM, Harrison JD, Butow PN, Solomon MJ, Masya L (2011). What is important in cancer care coordination? A qualitative investigation. Eur J Cancer Care.

[14] NSW Health. NSW Plan for Healthy Culturally and Linguistically Diverse Communities: 2019–2023 [Internet]. Sydney: NSW Health, 2019 https://www1.health.nsw.gov.au/pds/ActivePDSDocuments/PD2019_018.pdf. Accessed 16 Jul 2021.

[15] Watts KJ, Meiser B, Zilliacus E, Kaur R, Taouk M, Girgis A (2017). Communicating with patients from minority backgrounds: individual challenges experienced by oncology health professionals. Eur J Oncol Nurs.

[16] Watts KJ, Meiser B, Zilliacus E, Kaur R, Taouk M, Girgis A (2018). Perspectives of oncology nurses and oncologists regarding barriers to working with patients from a minority background: systemic issues and working with interpreters. Eur J Cancer Care..

[17] Henderson S, Kendall E (2011). Culturally and linguistically diverse peoples' knowledge of accessibility and utilisation of health services: exploring the need for improvement in health service delivery. Aust J Prim Health.

[18] Butow P, Bell M, Goldstein D, Sze M, Aldridge L, Abdo S (2011). Grappling with cultural differences; communication between oncologists and immigrant cancer patients with and without interpreters. Patient Educ Couns.

[19] Butow PN, Sze M, Dugal-Beri P, Mikhail M, Eisenbruch M, Jefford M (2010). From inside the bubble: migrants' perceptions of communication with the cancer team. Support Care Cancer.

[20] Sze M, Butow P, Bell M, Vaccaro L, Dong S, Eisenbruch M (2015). Migrant health in cancer: outcome disparities and the determinant role of migrant-specific variables. Oncologist.

[21] Komaric N, Bedford S, van Driel ML (2012). Two sides of the coin: patient and provider perceptions of health care delivery to patients from culturally and linguistically diverse backgrounds. BMC Health Serv Res.

[22] Licqurish S, Phillipson L, Chiang P, Walker J, Walter F, Emery J (2017). Cancer beliefs in ethnic minority populations: a review and meta-synthesis of qualitative studies. Eur J Cancer Care..

[23] Butow P, Baile WF. Communication in cancer care: a cultural perspective. In: Grassi L, Riba M, eds. Clinical Psycho‐Oncology; 2012. p. 11–20.

[24] Goldstein D, Bell ML, Butow P, Sze M, Vaccaro L, Dong S (2014). Immigrants' perceptions of the quality of their cancer care: an Australian comparative study, identifying potentially modifiable factors. Ann Oncol.

[25] Nic Giolla Easpaig B, Arnolda G, Tran Y, Bierbaum M, Lamprell K, Delaney GP (2019). What is multidisciplinary cancer care like in practice? A protocol for a mixed-method study to characterise ambulatory oncology services in the Australian public sector. BMJ Open..

[26] Charlesworth S, Baines D (2015). Understanding the negotiation of paid and unpaid care work in community services in cross-national perspective: the contribution of a rapid ethnographic approach. J Fam Stud.

[27] Millen, D. Rapid ethnography: time deepening strategies for HCI field research. Paper presented at: The 3rd Conference on Designing Interactive Systems: Processes, Practices, Methods, and Techniques, 2000, August, New York City, NY, United States.

[28] Australian Bureau of Statistics. Australian Bureau of Statistics 2016 Census QuickStats [Internet]. Canberra, ACT [updated 2017]. https://www.abs.gov.au/websitedbs/D3310114.nsf/Home/census. Accessed 1 Oct 2020.

[29] Nic Giolla Easpaig B, Tran Y, Arnolda G, Clay-Williams R, Delaney GP, Liauw W (2020). How do we work as researchers in the real world? Mapping the trajectory of methodological decision making in health services research. Int J Qual Methods..

[30] Phillips CB, Dwan K, Hepworth J, Pearce C, Hall S (2014). Using qualitative mixed methods to study small health care organizations while maximising trustworthiness and authenticity. BMC Health Serv Res.

[31] Joffe H, Harper D, Thompson AR (2011). Thematic analysis. Qualitative research methods in mental health and psychotherapy: a guide for students and practitioners.

[32] Cancer Australia. Principles for best practice management of lung cancer in Australia. Strawberry Hills, NSW. Cancer Australia, 2013. https://www.canceraustralia.gov.au/sites/default/files/publications/lcbp_principles_for_best_practice_management_of_lung_cancer_in_australia_52d60ed4c2a0d.pdf

[33] NSW Health. Interpreters – Standard Procedures for Working with Health Care Interpreters [Internet]. Sydney: NSW Health, 2017. https://www1.health.nsw.gov.au/pds/ActivePDSDocuments/PD2017_044.pdf. Accessed 16 Jul 2021.

[34] Elkaddoum R, Haddad FG, Eid R, Kourie HR (2020). Telemedicine for cancer patients during COVID-19 pandemic: between threats and opportunities. Future Oncol.

[35] Royce TJ, Sanoff HK, Rewari A (2020). Telemedicine for cancer care in the time of COVID-19. JAMA Oncol.

[36] Gany FM, Gonzalez CJ, Basu G, Hasan A, Mukherjee D, Datta M (2010). Reducing clinical errors in cancer education: interpreter training. J Cancer Educ.

[37] PricewaterhouseCoopers. Culturally and linguistically diverse patient costing study report. Independent Hospital Pricing Authority. 2015. https://www.ihpa.gov.au/sites/default/files/publications/culturally_and_linguistically_diverse_patient_costing_study.pdf

[38] Independent Hospital Pricing Authority. National Efficient Price Determination 2021–22 [Internet]. Sydney: Independent Hospital Pricing Authority, 2021. https://www.ihpa.gov.au/publications/national-efficient-price-determination-2021-22. Accessed 16 July 2021

[39] Mazza D, Lin X, Walter FM (2018). The LEAD study protocol: a mixed-method cohort study evaluating the lung cancer diagnostic and pre-treatment pathways of patients from Culturally and Linguistically Diverse (CALD) backgrounds compared to patients from Anglo-Australian backgrounds. BMC Cancer.

[40] Phillipson L, Larsen-Truong K, Jones S, Pitts, L. Improving cancer outcomes among culturally and linguistically diverse communities: a rapid review of the literature. Faculty of Social Sciences, University of Wollongong, Wollongong, Australia. 2012. https://ro.uow.edu.au/sspapers/486

