# Supplementary material for: Providing outpatient cancer care for CALD patients: a qualitative study
Source: BMC Res Notes. 2021 Aug 9;14:304. doi: 10.1186/s13104-021-05724-3 (PMC8350263; doi:10.1186/s13104-021-05724-3)
Supplement: Supplementary file 1 — Additional file 1. Interview topic guide. A topic guide for the interviews with key informants. [file 13104_2021_5724_MOESM1_ESM.docx]

**Additional file 1:** **Key Informant Interview Guide**

1. *Basic role and experience*

- Can you tell me about your role?
  - Typical activities, responsibilities, multidisciplinary team involvement? Services you work in?
- Professional background
  - How long have you been in the role?

1. *Models of care*

- Models of care currently used
  - Triage and risk categorization

1. *Patient journey*

- In your experience, are there critical times in the patient journey when engaging with supportive needs is especially important? What are these?

Discussion of the processes and practices involved in the patient journey will be mapping according to:

- Tumour groupings;
- The management of routine vs at risk or complex cases; and
- Service model for cancer care coordinator role (e.g. tumour-specific vs generic; privately vs publicly funded)
